# Early Life Stress, Physiology, and Genetics: A Review

**DOI:** 10.3389/fpsyg.2019.01668

**Published:** 2019-08-02

**Authors:** Nia Fogelman, Turhan Canli

**Affiliations:** Department of Psychology, Stony Brook University, Stony Brook, NY, United States

**Keywords:** early life stress, sympathetic nervous system, hypothalamic-pituitary-adrenal axis, inflammation, genetics, epigenetics

## Abstract

Early life stress (ELS) is a widely studied concept due to both its prevalent nature and its (presumed) detrimental consequences. In this review, we discuss the relationship between ELS and its underlying physiology spanning the sympathetic nervous system, hypothalamic-pituitary-adrenal axis, and markers of inflammation related to immune function in both human and animal literature. We also consider the potential role of genetic and epigenetic factors on the ELS-health outcome relationship. We conclude with recommendations to overcome identified shortcomings in a field that seeks to address the health consequences of ELS.

## Introduction

Early life stress (ELS), including physical, sexual, and emotional forms of abuse and neglect experienced by the developing child (Centers for Disease Control and Prevention, 2018), has been linked to a host of physical and psychological sequelae into adulthood (Chapman et al., 2004; Gluckman et al., 2008; Middlebrooks and Audage, 2008; Pechtel and Pizzagalli, 2011). Such adverse experiences are surprisingly common, according to a large-scale epidemiological study on adverse childhood events, which reports that approximately 65% of people in the United States experienced at least one, and 12.5% experienced as many as four, adverse early life events (Middlebrooks and Audage, 2008). Given these statistics, a deeper understanding of the pathophysiology of ELS could produce better long-term prognosticators of adverse sequelae for vulnerable individuals, and promote the development of patient-centered interventions with specific strategies to mitigate the ill effects of ELS.

To elucidate the connection between ELS and negative health outcomes, research examined putative physiological mediators, including the sympathetic nervous system (SNS), the hypothalamic pituitary adrenal (HPA) axis, and cytokines linked to inflammation (Mayer, 2000; Pariante and Lightman, 2008). Dysregulation of these systems has been associated with a host of disorders in and of themselves (Cohen et al., 2000; Cowen, 2010; Lamers et al., 2013). In this review, we will examine the connections between ELS and these physiological pathways in both human adults and mature non-human animals. We will additionally consider the potential role of candidate gene polymorphisms and DNA methylation in genes linked to the stress response and to mood disorders as moderators of the ELS-negative health outcome relationship. Finally, we will offer recommendations for a growing field examining the effects of ELS into adulthood.

## An Overview of Acute Stress Physiology and Inflammatory Markers

Stress promotes physiological responses across several different systems, most prominently the SNS, HPA axis, and immune system, which are heavily intertwined (Chrousos, 2009). For instance, brain regions processing sensory and psychological stressors activate preganglionic neurons in brain stem and spinal cord to activate the peripheral SNS. This then prepares the body to “fight or flee” by releasing catecholamines such as epinephrine and norepinephrine to increase heart rate and blood pressure. In parallel, stress activates the HPA axis, leading to the release of corticotropin-releasing factor (CRF) by the hypothalamus, followed by the release of adrenocorticotropin-releasing hormone (ACTH) onto CRH-R1 receptors from the pituitary, and subsequent release of glucocorticoids (including cortisol in humans and corticosterone in animals) from the adrenal glands (Lupien et al., 2009). Both activation of the SNS and HPA axis are correlated with inflammatory markers, including tumor necrosis factor-α (TNF-α), interferon-γ (IFN-γ), and interleukin-6 (IL-6) (Chrousos and Gold, 1992; Tsigos and Chrousos, 2002; Padgett and Glaser, 2003).

**Figure 1:**
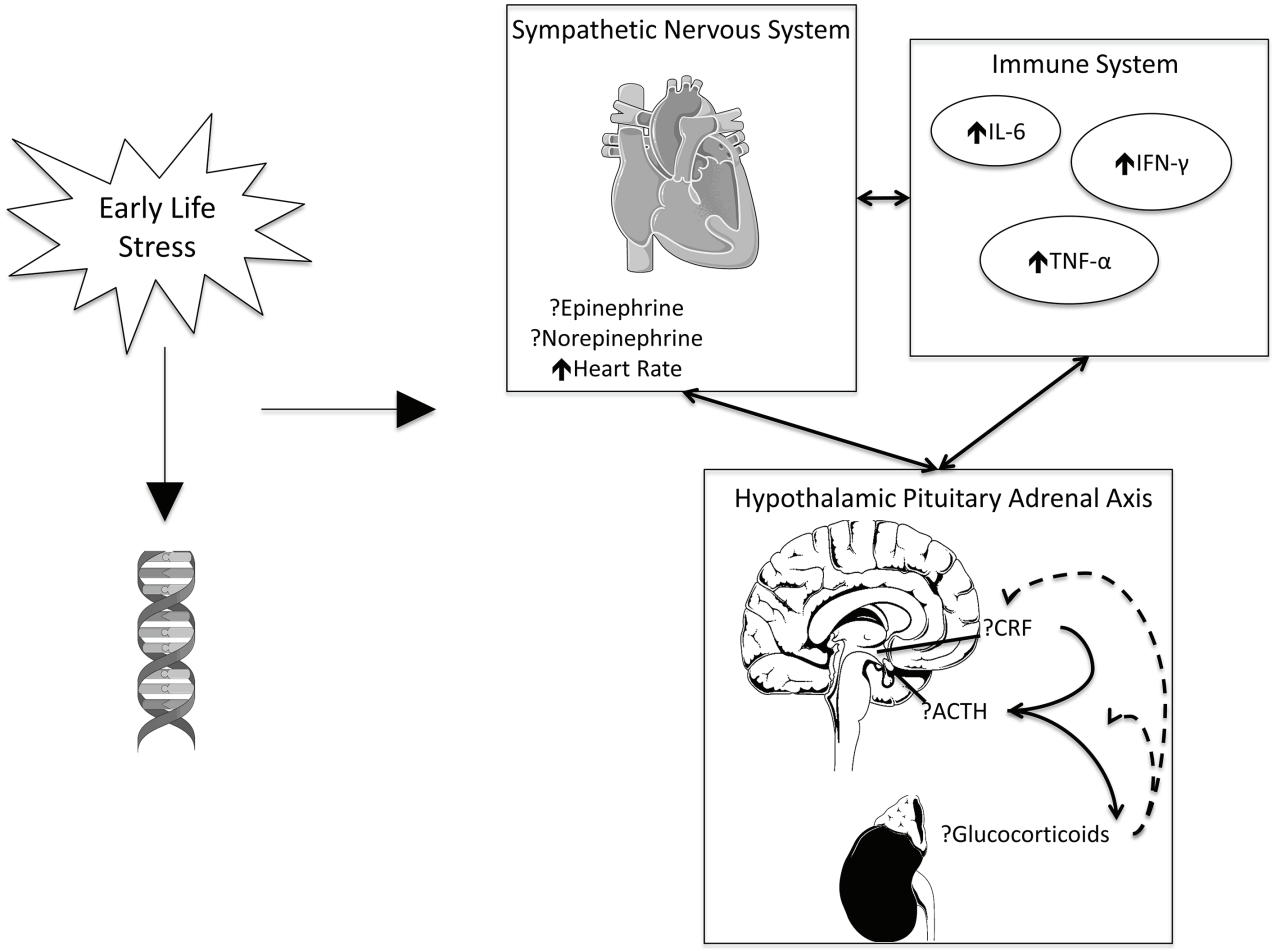
ELS may impact gene methylation. ELS, working in conjunction with genetics, is associated with increased heart rate and inflammation. Evidence is inconsistent on the effects of catecholamines and signaling pathways associated with the HPA axis. The SNS, HPA axis, and immune system exhibit reciprocal effects on one another. Images adapted from open license image site smart.servier.com.

For the purposes of this review, we will focus on these and similar markers to assess the impact of ELS on stress physiology. Figure 1 presents an overview of these markers, their relationship to one another, and their potential changes in the presence of stress.

## Early Life Stress, Sympathetic Nervous System, Hypothalamic-Pituitary-Adrenal Axis, and Inflammation in Humans

Several studies have found a relationship between ELS and cardiovascular/HPA axis effects. For instance, Dong et al. (2004) saw a direct relationship between ELS and ischemic heart disease later in life, although this was partially mediated by presence of additional psychological risk variables such as anger levels and depressed affect. Gatt et al. (2009) observed that an increase in the number of ELS events in healthy adult participants was associated with higher resting and activated heart rates. Similarly, Heim et al. (2000) reported elevated heart rate in response to the Trier Social Stress Test (TSST) in a group of depressed women who had experienced sexual and/or physical abuse early in life, compared to non-depressed women without ELS. Within the same group, depressed ELS women displayed heightened cortisol reactivity and dysregulated ACTH (the latter was seen in women with ELS regardless of depression status; Heim et al., 2000). In line with a heightened cortisol response, individuals in a community-based sample with ELS and a psychiatric history displayed poorer recovery and exhibited significantly stronger cortisol reactivity to a cognitive stress task, compared to the non-ELS group (Goldman-Mellor et al., 2012). Yet, others reported *diminished* cortisol associated with ELS. For instance, women who had experienced ELS showed lower baseline cortisol levels preceding a corticotropin-releasing factor (CRF) stimulation test (a bolus of CRF given in the afternoon), but produced a similar post-stressor cortisol response, compared to non-ELS controls (Heim et al., 2001). ELS was also associated with blunted cortisol in another common HPA outcome measure, the cortisol awakening response, in a different study (Li et al., 2015). Given the conflicting literature, we conducted a comprehensive meta-analysis, in which we found no significant association between early life stress and cortisol (Fogelman and Canli, 2018). We did, however, note strong heterogeneity across studies, suggesting that “type of stressor” may be an important moderator of subsequent cortisol physiology. For instance, experiencing abuse was associated with increased cortisol awakening response levels, suggesting that some forms of trauma may be more severe and hence have a more profound and lasting impact on physiology.

ELS is also associated with increased inflammation, both basally and in response to acute stress. One longitudinal study that followed participants since childhood, demonstrated a link between ELS and high-sensitivity C-reactive protein (hsCRP) levels (Danese et al., 2007). ELS was also associated with heightened IL-6 in response to the TSST (Carpenter et al., 2010) and with heightened IL-1β, IL-12, and TNF-α levels (Li et al., 2015) in healthy community samples. In a patient cohort of depressed individuals, there was no significant main effect between ELS and inflammatory markers; however, depressed patients displayed greater ELS and an increase in nuclear factor kappa-light-chain-enhancer of activated B cells (NF-κB) and IL-6 in response to the TSST (Pace et al., 2006).

In addition to ELS being linked to the SNS, HPA axis, and immune system, these systems have been shown to influence one another. For instance, Fagundes and Way (2014) proposed that increased sympathetic activity and dysregulated cortisol attributed to ELS may persist to low-grade inflammation over time. The “neuro-immune network” hypothesis by Nusslock and Miller (2016) puts forth the notion that ELS strengthens cortico-amygdala neural circuitry, thus “priming” and responding to heightened SNS, HPA axis, and immune function over time. Taken together, these findings suggest that ELS, at least in certain groups and under certain conditions, has a potent effect on mediating pathways related to the cardiovascular system/HPA axis and markers of inflammation.

## Early Life Stress, The Hypothalamic-Pituitary-Adrenal Axis, and Inflammation in Animals

In addition to studying the effects of ELS on health in human subjects, results from animal studies may offer helpful insights given the greater ability to manipulate experimental conditions and the simplified nature conditions surrounding ELS relative to human studies. Overall, experimental studies conducted in animal models of ELS tend to concur with human data suggesting heightened reactivity following ELS. For instance, one study found that rats who as pups had experienced maternal separation for either 15 or 180 min per day displayed higher levels of neuropeptide-y (a highly prevalent peptide associated with the CNS) than non-handled controls (Husum and Mathe, 2002), mirroring greater CNS reactivity we often see in human studies of ELS. In the same study, neuropeptide-y/CRF ratios were significantly lower in the maternal separation groups and the effect was dose dependent, such that more maternal separation was associated with lower ratios (Husum and Mathe, 2002); greater neuropeptide-y has been seen as protective in depression-like behavior in rats (Stogner and Holmes, 2000), therefore a lower ratio represents a concern for increased rates of depression-like behavior in the presence of ELS. Other maternal separation studies reported elevated heart rate and inflammatory responses to a physiological stressor (Loria et al., 2010), as well as elevated corticosterone, TNF-α, and IFN-γ levels, and increased production of anxiety-like behaviors (O’Mahony et al., 2009) in maternally deprived rats, compared to controls. In mice, ELS has also been linked with heightened basal corticosterone (Murgatroyd et al., 2009). These findings bolster concerns of up-regulation of physiological response, similar to that seen in human studies.

Yet, as with the human literature, reports on the impact of ELS on physiology are not always consistent. For instance, social isolation in early development was associated with *lower* corticosterone levels in a recovery period following restraint-stress exposure in adult rats (Lukkes et al., 2009). According to one review, rats and primates experiencing maternal separation early in life overall display heightened CRF, but diminished ACTH and glucocorticoid levels; in primates, these levels are initially elevated during development, suggesting that the timing of when measurements are obtained may be an important moderating factor (Pryce et al., 2002). On the whole, the animal literature mostly supports the conclusion that animal models of ELS produce later increases in SNS, HPA axis, and inflammatory markers. The larger degree of consistency across animal studies, compared to the human literature, may have been aided by greater experimental controls, more uniform models of ELS, and homogeneity in the choice of rodent strains.

## Early Life Stress and Negative Health Outcomes: The Role of Genetics

Thus far, we have reviewed evidence surrounding ELS and its relationship to the SNS, HPA axis, and immune function, as these may be pathways to negative health later in life. However, ELS may also lead to poorer health outcomes through genetic mechanisms. Indeed, numerous studies have considered the role of genetics, both at the structural level of gene variants and at the level of epigenetic regulation of DNA expression (Heim and Binder, 2012). Variants of well-known candidate genes may potentially increase susceptibility to stressful environmental conditions, increasing risk for mood and anxiety disorders (Nugent et al., 2011). In a seminal longitudinal study of approximately 1,000 individuals, Caspi et al. (2003) reported a significant interaction between presence of the serotonin-transporter-linked polymorphic region (5-HTTLPR) S allele and life stress history predicting depressive symptoms. Later replication studies produced conflicting results and meta-analyses came to opposite conclusions. One the one hand, one meta-analysis concluded that ELS significantly interacted with presence of the S allele in 5-HTTLPR of *SLC6A4* to predict greater stress sensitivity and risk of depression (Karg et al., 2011). On the other hand, a recent large-scale meta-analysis used harmonized analyses across 31 previously published datasets representing more than 38,000 individuals and failed to identify an interaction between the 5-HTTLPR S allele, life stress, and depression (Culverhouse et al., 2018). The authors concluded that “if an interaction exists in which the S allele of 5-HTTLPR increases risk of depression only in stressed individuals, then it is not broadly generalisable, but must be of modest effect size and only observable in limited situations” (p. 134).

Some other candidate genes included *FKBP5* (a co-chaperone to glucocorticoid receptors as part of the HPA axis leading to stress response regulation and anxiety); the gene encoding the brain-derived neurotrophic factor, *BDNF* (hypothesized role in mood disorders; Nugent et al., 2011); and the oxytocin receptor gene, *OXTR* (oxytocin receptors are concentrated in the hypothalamus and dysregulation has been associated with depression and anxiety). For example, Binder et al. (2008) identified four SNPs in the *FKBP5* gene interacting with ELS to predict heightened PTSD symptom severity; similar findings were reported by Klengel et al. (2013), however their results provided robust findings for only the rs1360780 SNP. Presence of the met allele in the val66met *BDNF* polymorphism in conjunction with ELS predicted greater depression and anxiety symptoms, although this was specifically mediated through diminished left prefrontal cortex brain volume and heightened heart rate (Gatt et al., 2009). The rs139832701 SNP in *OXTR* interacted with ELS to predict increased stress and depression scores (Myers et al., 2014).

Candidate gene studies have often been criticized for being statistically underpowered, and large-scale replication attempts have not supported previously reported gene-by-environment interactions. In addition to the Culverhouse et al. (2018) study, another recent large-state analysis imputed data (i.e., did not genotype directly, but inferred genotype from tagging single nucleotide polymorphisms) from large population-based and case-control samples (Ns ranging from 62,138 to 443,264 across subsamples) for 18 candidate genes (including *SLC6A4* and *BDNF,* but not *FKBP5* or *OXTR*) and also failed to find support for any main effect or interaction predicting depression (Border et al., 2019). These authors went further than Culverhouse et al. (2018), concluding “it is time for depression research to abandon historical candidate gene and candidate gene-by-environment interaction hypotheses” (p. 386).

The criticism of candidate genes raises important concerns about sample size and false positive results. Yet, it is also possible that analyses that are limited to DNA sequence variations and one specific disease outcome miss much of the complexity by which genes, life experience, and health outcomes are interrelated. For example, it is possible that additional mechanisms that have not been considered in the prior literature, such as alternative splicing of the human *SLC6A4* (Bradley and Blakely, 1997), contribute to inconsistent findings across study population. The effects of alternative splicing, as well as epigenetic mechanisms (discussed next), would be evident at the level of mRNA or protein expression, for which there are currently only limited datasets available.

The current literature on epigenetics and life stress suggests important mechanisms linking ELS to epigenetic gene regulatory mechanisms, particularly DNA methylation. DNA methylation is the process by which a methyl group binds to, most typically, Cytosin-Guanine (CpG) sites on DNA and regulates its expression (for further information see Moore et al., 2013). Such methylation may be caused by life stress. As early as *in utero*, experiences related to maternal nutrition and health can lead to an increased risk for metabolic dysregulation (e.g., in insulin and leptin) later in life with ELS-induced adaptations in DNA methylation as a proposed pathway to such a relationship (Gluckman et al., 2008). Therefore, examining how ELS is associated with methylation may provide insight into one pathway toward dysregulation.

Several studies have reported increased DNA methylation in the presence of ELS. First reported in rats in a seminal study by Weaver et al. (2004), poor maternal care was linked to increased methylation of the glucocorticoid receptor (GR) gene promoter, reduced GR expression in the hippocampus, and increased adult stress reactivity, which could be reversed with administration of the histone deacetylase (HDAC) inhibitor trichostatin A (TSA). A study by McGowan et al. (2009) extended these findings to humans, showing increased GR methylation and reduced GR mRNA expression in the hippocampus of suicide victims with a history of childhood abuse, compared to suicide victims without childhood abuse and non-suicide controls. In other work, adult participants who had experienced physical abuse as children had higher *SCL6A4* DNA methylation in blood relative to those who had not (Beach et al., 2010). Those with major depressive disorder (MDD) who had experienced ELS also exhibited a greater percentage of average methylation of *SLC6A4* in blood cells compared to those with MDD who had not experienced ELS (Kang et al., 2013). In those with PTSD, the ELS group had a greater proportion of transcripts with methylated CpG sites in blood cells compared to the no ELS group (Mehta et al., 2013). Evidence for ELS and methylation exists across species as well. Rats that experienced abusive behavior during development by stressed mothers showed increased DNA methylation of *BDNF* in the prefrontal cortex at exons IV and IX (Roth et al., 2009). Finally, the presence of methylation may be a function of both genotype and the presence of ELS. For instance, Duman and Canli (2015) found that presence of the S allele in the serotonin-transporter-linked polymorphic region (5-HTTLPR) interacted with ELS to predict greater average methylation of *SCL6A4* in blood samples; however, this did not correspond to downstream mRNA expression, suggesting more than just DNA methylation effects need to be considered. ELS interacted with rs1360780 in *FKBP5* such that presence of the T allele in conjunction with ELS was associated with decreased methylation percentage of intron 7 in blood cells (Klengel et al., 2013). This evidence collectively suggests that methylation may represent one pathway to poorer health.

## Conclusions and Future Recommendations

It is now widely accepted that ELS is linked to significant adverse sequelae in adulthood (Enoch, 2011; Shonkoff et al., 2012). However, this association needs to be better understood in its nuances. For instance, different types of ELS may be differentially associated with health risk; although most forms of ELS are associated with increased risk of psychopathology, surprisingly, this association was weaker for forms of physical neglect (Carr et al., 2013). This may stem from discrepancies in the severity of ELS types or from differences in the response (or lack thereof) to different ELS types. Negative consequences of ELS may be most pronounced when the stress takes on its most severe forms (Caspi et al., 2003; Kendler et al., 2004), when there are numerous instances of ELS (Heim et al., 2002; Dube et al., 2003; Myers et al., 2014), or in the context of other moderating factors. This includes, but is certainly not limited to, genetic disposition (Nugent et al., 2011) and ELS-related epigenetic modifications of genes involved in the stress systems (Heim and Binder, 2012).

Another consideration is that ELS perhaps does not present a unique threat to underlying physiology relative to other forms of stress. In one area of thought, ELS is unique because it takes place during developmentally sensitive periods, where underlying physiology later in life is particularly vulnerable to stressful experiences (Lupien et al., 2009). However, other evidence calls into doubt the singular role of ELS in that some forms of stress have a profound impact regardless of their timing. For example, sexual abuse, whether it occurs early in life or later on in adulthood, has been linked to a host of somatic disorders, including gastrointestinal problems and chronic pain (Paras et al., 2009). Similarly, an increase in stressful life events (regardless of age that the stressors occurred) was associated with increased depression (Risch et al., 2009) and evidence exists that such lifelong stress interacts with the presence of the S allele on 5-HTTLPR to predict increased rates of depression (Caspi et al., 2003), although this finding continues to generate debate (Karg et al., 2011; Culverhouse et al., 2018). Further complicating this narrative is the notion that different types of ELS co-occur (Merrick et al., 2018). Therefore, the detrimental effects of stress may not be restricted to the early developmental period, but rather might be determined by severity and number of different stressors.

Based on these considerations, we make three recommendations for the field to advance: (1) begin with rigorous operational definitions of ELS, thus making it easier to measure and compare findings across studies. At present, there are numerous methodologies to measure ELS (e.g., questionnaires (Bernstein et al., 1994), interview methods (Bremner et al., 2000), or by asking a social worker or caregiver if previous abuse has occurred), all potentially introducing unique variance that may help explain some of the inconsistent results. Showing correlations between instrument types within the same study (e.g., validating a social worker’s report with a high score on a standardized questionnaire by the trauma survivor) may set a gold standard to interpret any findings. (2) Consider ELS in a larger genetic and environmental context. This would span genetic profiles for multiple genes of interest or polygenic risk scores (for further information see Dudbridge, 2013), as well as current stressors in the person’s life and presence of social support (Heim et al., 2008; Daskalakis et al., 2013). (3) Conduct research in ways that mitigate, reframe, or reverse the consequences of ELS. Teaching children appropriate coping mechanisms and building social networks (Werner, 1995) may act as deterrents to these negative outcomes. Likewise, in applying lessons from the animal literature, environmental enrichment (i.e., cognitive stimulation and physical activity) may act as another mechanism to help mitigate negative effects (Fox et al., 2006).

## Author Contributions

NF wrote the initial draft. TC revised the initial draft. Both TC and NF conceptualized the material for review.

### Conflict of Interest Statement

The authors declare that the research was conducted in the absence of any commercial or financial relationships that could be construed as a potential conflict of interest.

## References

[ref1] Beach S. R. H. Brody G. H. Todorov A. A. Gunter T. D. Philibert R. A. (2010). Methylation at SLC6A4 is linked to family history of child abuse: an examination of the Iowa adoptee sample. Am. J. Med. Genet. B Neuropsychiatr. Genet. 153B, 710–713. 10.1002/ajmg.b.31028, PMID: 19739105 PMC2909112

[ref2] Bernstein D. P. Fink L. Handelsman L. Foote J. Lovejoy M. Wenzel K.. (1994). Initial reliability and validity of a new retrospective measure of child abuse and neglect. Am. J. Psychiatry 151, 1132–1136. 10.1176/ajp.151.8.1132, PMID: 8037246

[ref3] Binder E. B. Bradley R. G. Liu W. Epstein M. P. Deveau T. C. Mercer K. B.. (2008). Association of FKBP5 polymorphisms and childhood abuse with risk of posttraumatic stress disorder symptoms in adults. JAMA 299, 1291–1305. 10.1001/jama.299.11.1291, PMID: 18349090 PMC2441757

[ref4] Border R. Johnson E. C. Evans L. M. Smolen A. Berley N. Sullivan P. F.. (2019). No support for historical candidate gene or candidate gene-by-interaction hypotheses for major depression across multiple large samples. Am. J. Psychiatry 176, 376–387. 10.1176/appi.ajp.2018.18070881, PMID: 30845820 PMC6548317

[ref5] Bradley C. C. Blakely R. D. (1997). Alternative splicing of the human serotonin transporter gene. J. Neurochem. 69, 1356–1367. 10.1046/j.1471-4159.1997.69041356.x, PMID: 9326263

[ref6] Bremner J. D. Vermetten E. Mazure C. M. (2000). Development and preliminary psychometric properties of an instrument for the measurement of childhood trauma: the early trauma inventory. Depress. Anxiety 12, 1–12. 10.1002/1520-6394(2000)12:1<1::AID-DA1>3.0.CO;2-W, PMID: 10999240

[ref7] Carpenter L. L. Gawuga C. E. Tyrka A. R. Lee J. K. Anderson G. M. Price L. H. (2010). Association between plasma IL-6 response to acute stress and early-life adversity in healthy adults. Neuropsychopharmacology 35, 2617–2623. 10.1038/npp.2010.159, PMID: 20881945 PMC2978751

[ref8] Carr C. P. Martins C. M. Stingel A. M. Lemgruber V. B. Juruena M. F. (2013). The role of early life stress in adult psychiatric disorders: a systematic review according to childhood trauma subtypes. J. Nerv. Ment. Dis. 201, 1007–1020. 10.1097/NMD.0000000000000049, PMID: 24284634

[ref9] Caspi A. Sugden K. Moffitt T. E. Taylor A. Craig I. W. Harrington H.. (2003). Influence of life stress on depression: moderation by a polymorphism in the 5-HTT gene. Science 301, 386–389. 10.1126/science.1083968, PMID: 12869766

[ref10] Centers for Disease Control and Prevention (2018). Child abuse and neglect: Definitions. Available at: https://www.cdc.gov/volenceprevention/childabuseandneglect/definitions.html (Accessed September 11, 2018).

[ref11] Chapman D. P. Whitfield C. L. Felitti V. J. Dube S. R. Edwards V. J. Anda R. F. (2004). Adverse childhood experiences and the risk of depressive disorders in adulthood. J. Affect. Disord. 82, 217–225. 10.1016/j.jad.2003.12.013, PMID: 15488250

[ref12] Chrousos G. P. (2009). Stress and disorders of the stress system. Nat. Rev. Endocrinol. 5, 374–381. 10.1038/nrendo.2009.106, PMID: 19488073

[ref13] Chrousos G. P. Gold P. W. (1992). The concepts of stress and stress system disorders. Overview of physical and behavioral homeostasis. JAMA 267, 1244–1252. 10.1001/jama.1992.03480090092034, PMID: 1538563

[ref14] Cohen H. Benjamin J. Geva A. B. Matar M. A. Kaplan Z. Kotler M. (2000). Autonomic dysregulation in panic disorder and in post-traumatic stress disorder: application of power spectrum analysis of heart rate variability at rest and in response to recollection of trauma or panic attacks. Psychiatry Res. 96, 1–13. 10.1016/S0165-1781(00)00195-5, PMID: 10980322

[ref15] Cowen P. J. (2010). Not fade away: the HPA axis and depression. Psychol. Med. 40, 1–4. 10.1017/S0033291709005558, PMID: 19335939

[ref16] Culverhouse R. C. Saccone N. L. Horton A. C. Ma Y. Anstey K. J. Banaschewski T.. (2018). Collaborative meta-analysis finds no evidence of a strong interaction between stress and 5-HTTLPR genotype contributing to the development of depression. Mol. Psychiatry 23, 133–142. 10.1038/mp.2017.44, PMID: 28373689 PMC5628077

[ref17] Danese A. Pariante C. M. Caspi A. Taylor A. Poulton R. (2007). Childhood maltreatment predicts adult inflammation in a life-course study. Proc. Natl. Acad. Sci. USA 104, 1319–1324. 10.1073/pnas.0610362104 17229839 PMC1783123

[ref18] Daskalakis N. P. Bagot R. C. Parker K. J. Vinkers C. H. De Kloet E. R. (2013). The three-hit concept of vulnerability and resilience: toward understanding adaptation to early-life adversity outcome. Psychoneuroendocrinology 38, 1858–1873. 10.1016/j.psyneuen.2013.06.008, PMID: 23838101 PMC3773020

[ref19] Dong M. Giles W. H. Felitti V. J. Dube S. R. Williams J. E. Chapman D. P.. (2004). Insights into causal pathways for ischemic heart disease: adverse childhood experiences study. Circulation 110, 1761–1766. 10.1161/01.CIR.0000143074.54995.7F, PMID: 15381652

[ref20] Dube S. R. Felitti V. J. Dong M. Chapman D. P. Giles W. H. Anda R. F. (2003). Childhood abuse, neglect, and household dysfunction and the risk of illicit drug use: the adverse childhood experiences study. Pediatrics 111, 564–572. 10.1542/peds.111.3.564, PMID: 12612237

[ref21] Dudbridge F. (2013). Power and predictive accuracy of polygenic risk scores. PLoS Genet. 9:e1003348. 10.1371/annotation/b91ba224-10be-409d-93f4-7423d502cba0, PMID: 23555274 PMC3605113

[ref22] Duman E. A. Canli T. (2015). Influence of life stress, 5-HTTLPR genotype, and SLC6A4 methylation on gene expression and stress response in healthy Caucasian males. Biol. Mood Anxiety Disord. 5:2. 10.1186/s13587-015-0017-x, PMID: 25995833 PMC4438516

[ref23] Enoch M. A. (2011). The role of early life stress as a predictor for alcohol and drug dependence. Psychopharmacology 214, 17–31. 10.1007/s00213-010-1916-6, PMID: 20596857 PMC3005022

[ref24] Fagundes C. P. Way B. (2014). Early-life stress and adult inflammation. Curr. Dir. Psychol. Sci. 23, 277–283. 10.1177/0963721414535603

[ref25] Fogelman N. Canli T. (2018). Early life stress and cortisol: a meta-analysis. Horm. Behav. 98, 63–76. 10.1016/j.yhbeh.2017.12.014, PMID: 29289660

[ref26] Fox C. Merali Z. Harrison C. (2006). Therapeutic and protective effect of environmental enrichment against psychogenic and neurogenic stress. Behav. Brain Res. 175, 1–8. 10.1016/j.bbr.2006.08.016, PMID: 16970997

[ref27] Gatt J. M. Nemeroff C. B. Dobson-Stone C. Paul R. H. Bryant R. A. Schofield P. R. (2009). Interactions between BDNF Val66Met polymorphism and early life stress predict brain and arousal pathways to syndromal depression and anxiety. Mol. Psychiatry 14, 681–695. 10.1038/mp.2008.143 19153574

[ref28] Gluckman P. D. Hanson M. A. Cooper C. Thornburg K. L. (2008). Effect of in utero and early-life conditions on adult health and disease. N. Engl. J. Med. 359, 61–73. 10.1056/NEJMra0708473, PMID: 18596274 PMC3923653

[ref29] Goldman-Mellor S. Hamer M. Steptoe A. (2012). Early-life stress and recurrent psychological distress over the lifecourse predict divergent cortisol reactivity patterns in adulthood. Psychoneuroendocrinology 37, 1755–1768. 10.1016/j.psyneuen.2012.03.010, PMID: 22475549

[ref30] Heim C. Binder E. B. (2012). Current research trends in early life stress and depression: review of human studies on sensitive periods, gene-environment interactions, and epigenetics. Exp. Neurol. 233, 102–111. 10.1016/j.expneurol.2011.10.032, PMID: 22101006

[ref31] Heim C. Newport D. J. Bonsall R. Miller A. H. Nemeroff C. B. (2001). Altered pituitary-adrenal axis responses to provocative challenge tests in adult survivors of childhood abuse. Am. J. Psychiatry 158, 575–581. 10.1176/appi.ajp.158.4.575, PMID: 11282691

[ref32] Heim C. Newport D. J. Heit S. Graham Y. P. Wilcox M. Bonsall R.. (2000). Pituitary-adrenal and autonomic responses to stress in women after sexual and physical abuse in childhood. JAMA 284, 592–597. 10.1001/jama.284.5.592, PMID: 10918705

[ref33] Heim C. Newport D. J. Mletzko T. Miller A. H. Nemeroff C. B. (2008). The link between childhood trauma and depressio: insights from HPA axis studies in humans. Psychoneuroendocrinology 33, 693–710. 10.1016/j.psyneuen.2008.03.008, PMID: 18602762

[ref34] Heim C. Newport D. J. Wagner D. Wilcox M. M. Miller A. H. Nemeroff C. B. (2002). The role of early adverse experience and adulthood stress in the prediction of neuroendocrine stress reactivity in women: a multiple regression analysis. Depress. Anxiety 15, 117–125. 10.1002/da.10015, PMID: 12001180

[ref35] Husum H. Mathe A. A. (2002). Early life stress changes concentrations of neuropeptide Y and corticotropin-releasing hormone in adult rat brain. Lithium treatment modifies these changes. Neuropsychopharmacology 27, 756–764. 10.1016/S0893-133X(02)00363-9, PMID: 12431850

[ref36] Kang H. J. Kim J. M. Stewart R. Kim S. Y. Bae K. Y. Kim S. W. (2013). Association of SLC6A4 methylation with early adversity, characteristics and outcomes in depression. Prog. Neuro-Psychopharmacol. Biol. Psychiatry 44, 23–28. 10.1016/j.pnpbp.2013.01.006 23333376

[ref37] Karg K. Burmeister M. Shedden K. Sen S. (2011). The serotonin transporter promoter variant (5-HTTLPR), stress, and depression meta-analysis revisited: evidence of genetic moderation. Arch. Gen. Psychiatry 68, 444–454. 10.1001/archgenpsychiatry.2010.189, PMID: 21199959 PMC3740203

[ref38] Kendler K. S. Kuhn J. W. Prescott C. A. (2004). Childhood sexual abuse, stressful life events and risk for major depression in women. Psychol. Med. 34, 1475–1482. 10.1017/S003329170400265X, PMID: 15724878

[ref39] Klengel T. Mehta D. Anacker C. Rex-Haffner M. Pruessner J. C. Pariante C. M.. (2013). Allele-specific FKBP5 DNA demethylation mediates gene-childhood trauma interactions. Nat. Neurosci. 16, 33–41. 10.1038/nn.3275, PMID: 23201972 PMC4136922

[ref40] Lamers F. Vogelzangs N. Merikangas K. R. De Jonge P. Beekman A. T. Penninx B. W. (2013). Evidence for a differential role of HPA-axis function, inflammation and metabolic syndrome in melancholic versus atypical depression. Mol. Psychiatry 18, 692–699. 10.1038/mp.2012.144, PMID: 23089630

[ref41] Li L. Chassan R. A. Bruer E. H. Gower B. A. Shelton R. C. (2015). Childhood maltreatment increases the risk for visceral obesity. Obesity 23, 1625–1632. 10.1002/oby.21143, PMID: 26146933 PMC4509989

[ref42] Loria A. S. Pollock D. M. Pollock J. S. (2010). Early life stress sensitizes rats to angiotensin II-induced hypertension and vascular inflammation in adult life. Hypertension 55, 494–499. 10.1161/HYPERTENSIONAHA.109.145391, PMID: 20026758 PMC2829259

[ref43] Lukkes J. L. Mokin M. V. Scholl J. L. Forster G. L. (2009). Adult rats exposed to early-life social isolation exhibit increased anxiety and conditioned fear behavior, and altered hormonal stress responses. Horm. Behav. 55, 248–256. 10.1016/j.yhbeh.2008.10.014, PMID: 19027017

[ref44] Lupien S. J. McEwen B. S. Gunnar M. R. Heim C. (2009). Effects of stress throughout the lifespan on the brain, behaviour and cognition. Nat. Rev. Neurosci. 10, 434–445. 10.1038/nrn2639, PMID: 19401723

[ref45] Mayer E. A. (2000). The neurobiology of stress and gastrointestinal disease. Gut 47, 861–869. 10.1136/gut.47.6.861, PMID: 11076888 PMC1728136

[ref46] McGowan P. O. Sasaki A. D’alessio A. C. Dymov S. Labonte B. Szyf M.. (2009). Epigenetic regulation of the glucocorticoid receptor in human brain associates with childhood abuse. Nat. Neurosci. 12, 342–348. 10.1038/nn.2270, PMID: 19234457 PMC2944040

[ref47] Mehta D. Klengel T. Conneely K. N. Smith A. K. Altmann A. Pace T. W. (2013). Childhood maltreatment is associated with distinct genomic and epigenetic profiles in posttraumatic stress disorder. Proc. Natl. Acad. Sci. USA 110, 8302–8307. 10.1073/pnas.1217750110 23630272 PMC3657772

[ref48] Merrick M. T. Ford D. C. Ports K. A. Guinn A. S. (2018). Prevalence of adverse childhood experiences from the 2011–2014 behavioral risk factor surveillance system in 23 states. JAMA Pediatr. 172, 1038–1044. 10.1001/jamapediatrics.2018.2537, PMID: 30242348 PMC6248156

[ref49] Middlebrooks J. S. Audage N. C. (2008). “The effects of childhood stress on health across the lifespan” in National center for injury prevention and control of the centers for disease control and prevention. National Center for Injury Prevention and Control (U.S.). Atlanta, GA: Centers for Disease Control and Prevention.

[ref50] Moore L. D. Le T. Fan G. (2013). DNA methylation and its basic function. Neuropsychopharmacology 38, 23–38. 10.1038/npp.2012.112, PMID: 22781841 PMC3521964

[ref500] Murgatroyd C. Patchev A. V. Wu Y. Micale V. Bockmühl Y. Fischer D.. (2009). Dynamic DNA methylation programs persistent adverse effects of early-life stress. Nat. Neuro. 12, 1559–1566. 10.1038/nn.2436, PMID: 19898468

[ref51] Myers A. J. Williams L. Gatt J. M. McAuley-Clark E. Z. Dobson-Stone C. Schofield P. R.. (2014). Variation in the oxytocin receptor gene is associated with increased risk for anxiety, stress and depression in individuals with a history of exposure to early life stress. J. Psychiatr. Res. 59, 93–100. 10.1016/j.jpsychires.2014.08.021, PMID: 25262417 PMC4252971

[ref52] Nugent N. R. Tyrka A. R. Carpenter L. L. Price L. H. (2011). Gene-environment interactions: early life stress and risk for depressive and anxiety disorders. Psychopharmacology 214, 175–196. 10.1007/s00213-010-2151-x, PMID: 21225419 PMC3615637

[ref53] Nusslock R. Miller G. E. (2016). Early-life adversity and physical and emotional health across the lifespan: a neuroimmune network hypothesis. Biol. Psychiatry 80, 23–32. 10.1016/j.biopsych.2015.05.017, PMID: 26166230 PMC4670279

[ref54] O’Mahony S. M. Marchesi J. R. Scully P. Codling C. Ceolho A. M. Quigley E. M.. (2009). Early life stress alters behavior, immunity, and microbiota in rats: implications for irritable bowel syndrome and psychiatric illnesses. Biol. Psychiatry 65, 263–267. 10.1016/j.biopsych.2008.06.026, PMID: 18723164

[ref55] Pace T. W. Mletzko T. C. Alagbe O. Musselman D. L. Nemeroff C. B. Miller A. H.. (2006). Increased stress-induced inflammatory responses in male patients with major depression and increased early life stress. Am. J. Psychiatry 163, 1630–1633. 10.1176/ajp.2006.163.9.1630, PMID: 16946190

[ref56] Padgett D. A. Glaser R. (2003). How stress influences the immune response. Trends Immunol. 24, 444–448. 10.1016/S1471-4906(03)00173-X, PMID: 12909458

[ref57] Paras M. L. Murad M. H. Chen L. P. Goranson E. N. Sattler A. L. Colbenson K. M.. (2009). Sexual abuse and lifetime diagnosis of somatic disorders: a systematic review and meta-analysis. JAMA 302, 550–561. 10.1001/jama.2009.1091, PMID: 19654389

[ref58] Pariante C. M. Lightman S. L. (2008). The HPA axis in major depression: classical theories and new developments. Trends Neurosci. 31, 464–468. 10.1016/j.tins.2008.06.006, PMID: 18675469

[ref59] Pechtel P. Pizzagalli D. A. (2011). Effects of early life stress on cognitive and affective function: an integrated review of human literature. Psychopharmacology 214, 55–70. 10.1007/s00213-010-2009-2, PMID: 20865251 PMC3050094

[ref60] Pryce C. R. Ruedi-Bettschen D. Dettling A. C. Feldon J. (2002). Early life stress: long-term physiological impact in rodents and primates. News Physiol. Sci. 17, 150–155. 10.1152/nips.01367.2001. PMID: 12136043

[ref61] Risch N. Herrell R. Lehner T. Liang K. Y. Eaves L. Hoh J.. (2009). Interaction between the serotonin transporter gene (5-HTTLPR), stressful life events, and risk of depression: a meta-analysis. JAMA 301, 2462–2471. 10.1001/jama.2009.878, PMID: 19531786 PMC2938776

[ref62] Roth T. L. Lubin F. D. Funk A. J. Sweatt J. D. (2009). Lasting epigenetic influence of early-life adversity on the BDNF gene. Biol. Psychiatry 65, 760–769. 10.1016/j.biopsych.2008.11.028, PMID: 19150054 PMC3056389

[ref63] Shonkoff J. P. Garner A. S. Committee on Psychosocial Aspects Of, C., Family, H., Committee on Early Childhood, A., Dependent, C., Section On, D., and Behavioral, P (2012). The lifelong effects of early childhood adversity and toxic stress. Pediatrics 129, e232–e246. 10.1542/peds.2011-2663 22201156

[ref64] Stogner K. A. Holmes P. V. (2000). Neuropeptide-Y exerts antidepressant-like effects in the forced swim test in rats. Eur. J. Pharmacol. 387, R9–R10. 10.1016/S0014-2999(99)00800-6, PMID: 10650166

[ref65] Tsigos C. Chrousos G. P. (2002). Hypothalamic-pituitary-adrenal axis, neuroendocrine factors and stress. J. Psychosom. Res. 53, 865–871. 10.1016/S0022-3999(02)00429-4, PMID: 12377295

[ref66] Weaver I. C. Cervoni N. Champagne F. A. D’alessio A. C. Sharma S. Seckl J. R.. (2004). Epigenetic programming by maternal behavior. Nat. Neurosci. 7, 847–854. 10.1038/nn1276, PMID: 15220929

[ref67] Werner E. E. (1995). Resilience in development. Curr. Dir. Psychol. Sci. 4, 81–84.

